# Burned bodies: post-mortem computed tomography, an essential tool for modern forensic medicine

**DOI:** 10.1007/s13244-018-0633-2

**Published:** 2018-06-07

**Authors:** J.-B. Coty, C. Nedelcu, S. Yahya, V. Dupont, C. Rougé-Maillart, M. Verschoore, C. Ridereau Zins, C. Aubé

**Affiliations:** 1 Department of Radiology, University Hospital of Angers, Medicine University of Angers, 4 rue Larrey, 49933, Cedex 9 Angers, France; 2 Department of Radiology, University Hospital of Angers, Angers, France; 3 Department of Forensic Medicine, University Hospital of Angers, Angers, France; 4 Department of Forensic Medicine, University Hospital of Angers, Medicine University of Angers, Angers, France; 5 Direction Générale, University Hospital of Angers, Angers, France

**Keywords:** Post-mortem computed tomography, Burned bodies, Thermal epidural haematoma, Thermal amputation

## Abstract

**Abstract:**

Currently, post-mortem computed tomography (PMCT) has become an accessible and contemporary tool for forensic investigations. In the case of burn victims, it provides specific semiologies requiring a prudent understanding to differentiate between the normal post-mortem changes from heat-related changes. The aim of this pictorial essay is to provide to the radiologist the keys to establish complete and focused reports in cases of PMCT of burn victims. Thus, the radiologist must discern all the contextual divergences with the forensic history, and must be able to report all the relevant elements to answer to the forensic pathologist the following questions: Are there tomographic features that could help to identify the victim? Is there evidence of remains of biological fluids in liquid form available for toxicological analysis and DNA sampling? Is there another obvious cause of death than heat-related lesions, especially metallic foreign bodies of ballistic origin? Finally, what are the characteristic burn-related injuries seen on the corpse that should be sought during the autopsy?

**Teaching points:**

*• CT is highly useful to find features permitting the identification of a severely burned body.*

*• PMCT is a major asset in gunshot injuries to depict ballistic foreign bodies in the burned cadavers.*

*• CT is able to recognise accessible blood for tests versus heat clot (air-crescent sign).*

*• Heat-related fractures are easily differentiated from traumatic fractures.*

*• Epidural collections with a subdural appearance are typical heat-related head lesions.*

## Introduction

Fire situations (domestic fire, arson, etc.) are current and may involve human victims.

In such cases, authorities initiate a judicial inquiry and mandate the forensic department to undertake investigation and exclude an arson attack.

Although the charred bodies are generally not completely destroyed, it remains a difficult and challenging task for the forensic physician who is expected to determine: the identity of the victim, the presence or absence of essential signs that could indicate whether the deceased was alive or not when the fire broke out, the cause of death, potential poisoning or intoxication (carbon monoxide, alcohol, drugs, etc.), the possibility of a third party intervention and of potential criminal involvement.

**Fig. 1:**
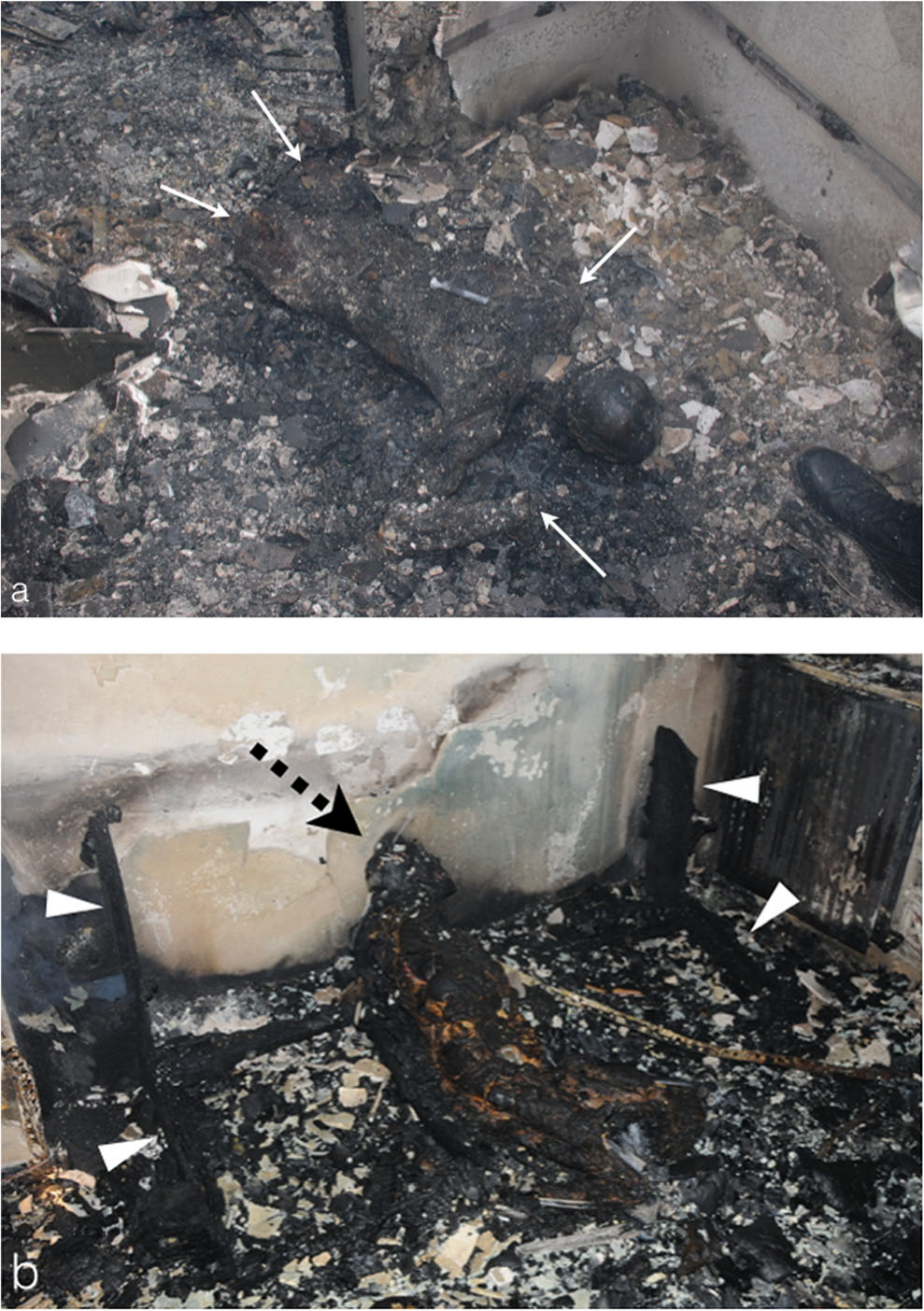
Forensic photographic images taken prior to the removal of charred bodies in home fire debris. **a** The charred body of a 32-year-old man who died by carbon monoxide poisoning in his burned home. The body was found by firefighters in the procubitus position (as shown) on the bathroom floor. This position suggests that the victim tried to escape from the fire by crawling on the floor. Note the severe skin burn injuries with thermal amputation of the limbs (*white arrows*). **b** Burned corpse of a 36-year-old man, found by firefighters in a suspicious sitting position (*black dashed arrow*) on the remains of a sofa (*white arrowheads*). The position of the body suggests no attempt to escape the fire, in keeping with the hypothesis that death occurred before the fire broke out. The autopsy showed a linear wound of the pulmonary artery and the aorta, in relation to a stab injury (not shown here) and hence suggesting an arson to cover up an underlying homicide

Primarily, the forensic team starts by collecting relevant information during the removal of corpses, such as the position of the body in the fire debris (Fig. 1), its temperature and its carbonisation degree.

Subsequently, the forensic pathologist will carry out specific toxicological screening tests for blood levels of substances such as carbon monoxide and cyanide, to determine whether or not the victim was alive when the fire was initiated.

Finally, considerable reliance can be laid upon the various imaging techniques to help the forensic pathologist in the victim’s identification process, and to guide him in the autopsy, fluid analysis and DNA sampling. Indeed, the advanced state of carbonisation often complicates the surgical dissection and some foreign bodies (bullets, prostheses, etc.) or bone alterations (osteosynthesis, traumatic fractures, etc.) could be missed.

Thus, via the modernisation and improvement of cross-sectional imaging techniques, post-mortem computed tomography (PMCT) has superseded conventional radiography and can provide an entire-body volumetric exploration to help the forensic team in their investigations [1, 2].

This imaging modality is nowadays accessible, reproducible, reliable and easy to implement.

Undoubtedly, contrary to the other post-mortem situations, there is no discussion regarding the use of contrast enhancement in the circumstance of severe burn victims; the severe skin and soft tissue injuries formally prevent the catheterisation of the blood vessels and render the injection of contrast agents impossible.

**Fig. 2:**
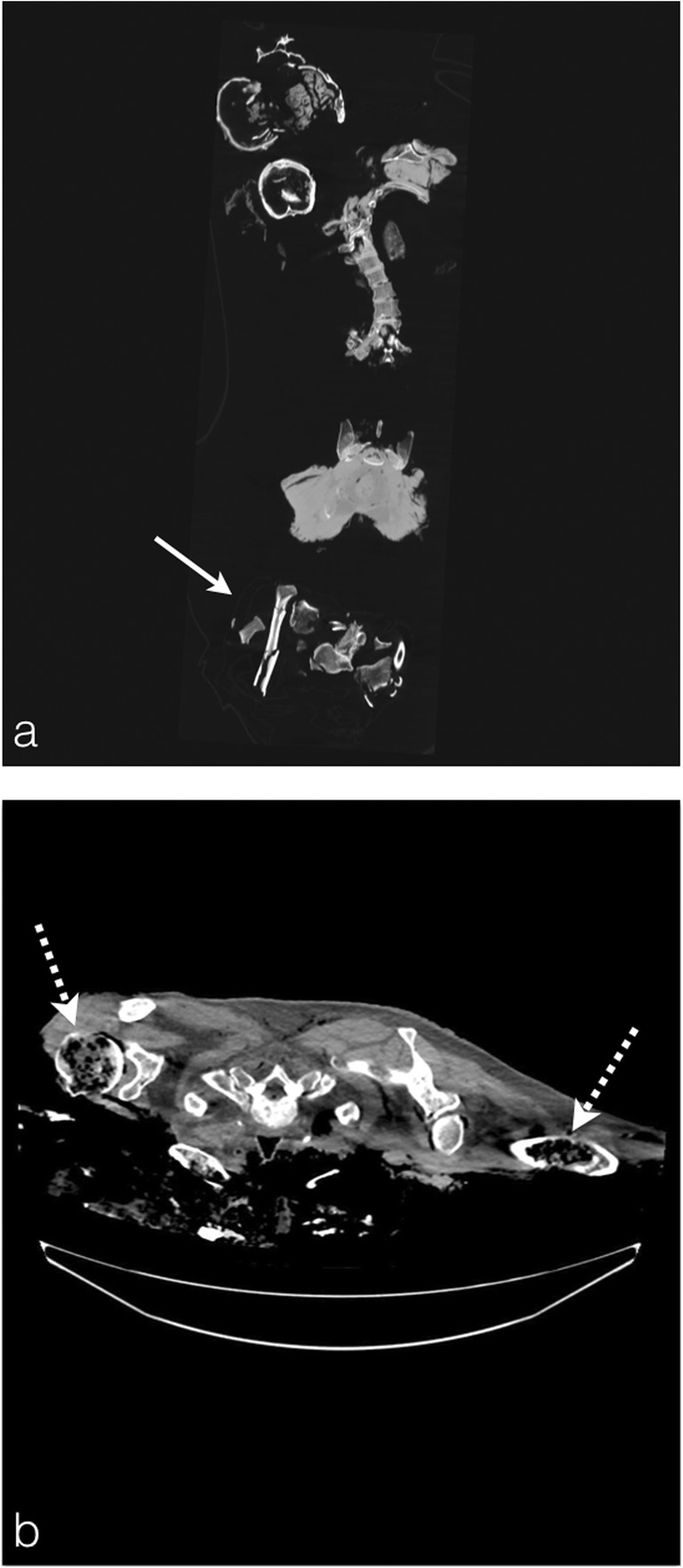
PMCT technical difficulties with burned bodies. **a** Coronal plane reconstruction of the charred body of a 58-year-old man found deceased in his burned car (suicide). Note the important tissue loss of this severely burned body with multiple bone fragments brought in a separate cadaver pouch for the CT scan (*white arrow*). In this difficult case, the victim’s body fragments must be differentiated from foreign-body contamination during the corpse removal process. **b** An 89-year-old man, killed in an accidental fire initiated from a miscontrolled fireplace at his home. The body contours were difficult to appreciate through the cadaver pouch and the burned body was initially put in a wrong position (procubitus) on the CT table. Note the extensive burn lesions of the directly fire-exposed bones conveying marked mottled lucencies in the marrow bone spaces (*white dashed arrow*)

Hence, the protocol is always a non-contrast-enhanced full-body exploration, and technical difficulties only remain in relation to the state of dilapidation of the charred body and its transport and installation on the CT table (Fig. 2).

The PMCT of a burned corpse provides specific imaging semiologies due to the presence of multiple heat-related body changes [3]. It requires a sagacious approach in order to differentiate between normal post-mortem changes from heat-related changes [4].

Finally, the aim of this pictorial essay is to provide to the radiologist the keys to establish complete and focused reports in the case of PMCT exploration of burn victims, and to respond to the main inquiries of the forensic pathologist, which include: Are there relevant elements that could help identify the victim? Are there possible sites for fluid analysis or DNA sampling? Is there another obvious cause of death other than heat-related lesions? What are the characteristic burn-related injuries seen on the corpse?

## Identification

The identification of the victim is one of the first duties requested by authorities to the forensic coroner. Sometimes, the victim’s burn injuries are minor and superficial, and identification remains feasible by the relatives. Nonetheless, in the majority of burn casualties, corpses are extensively charred and their identification is quite a challenge for the forensic team.

**Fig. 3:**
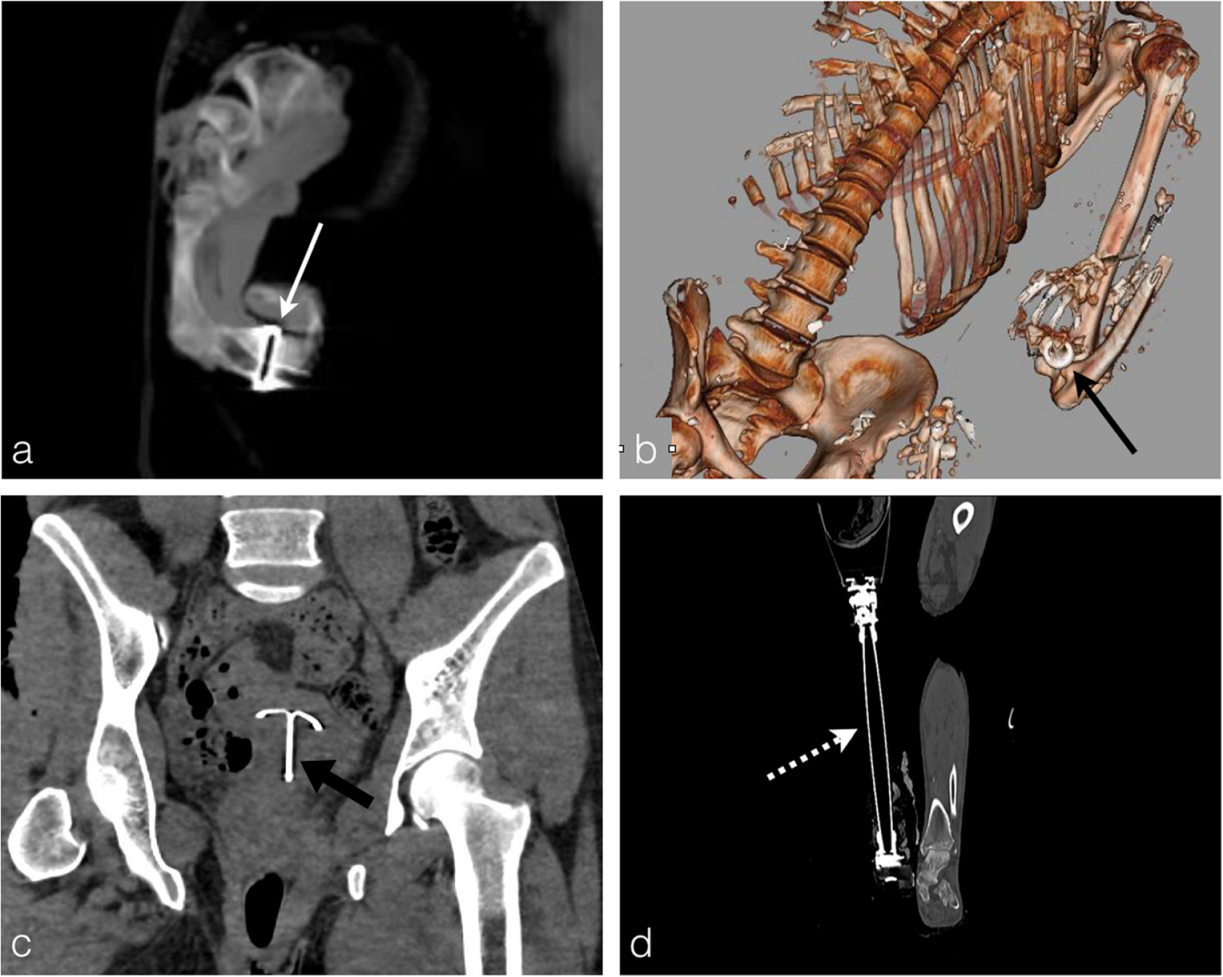
In the case of an extremely charred body, the foreign bodies viewable on PMCT could help the forensic team to identify the body and should be enumerated in the CT report. **a** Charred body of a 72-year-old man found under the debris from his home fire. Notice the metallic artefact of the signet ring on the wedding finger on MIP sagittal reconstruction (*white arrows*). **b** Volume rendering reconstruction of the same body with the ring around the wedding finger (*black arrow*). **c** The burned body of a 42-year-old woman, killed and then burned by her husband. The PMCT shows an intra-uterine device (*thick black arrow*). The presence of such medical devices is correlated with medical records by the forensic pathologist, to facilitate the process of victim identification. **d** The burned body of an 84-year-old man, who died from carbon monoxide poisoning in a home fire. Formal identification of the body was possible via research through medical records for correlation of right leg amputation with leg prothesis (*dashed white arrow*)

External elements of identification can be collected by macroscopic examination of the body (jewellery, watches, tattoos, etc.), as well as metallic objects which are highly radio-opaque and thus easily seen on the PMCT. Internal medical devices are useful to be reported in order to correlate them with the medical record of the alleged victim: vascular prothesis, osteosynthesis equipment, dental fillings, surgical clips, pace-maker, intra-uterine device, etc. (Fig. 3).

**Fig. 4:**
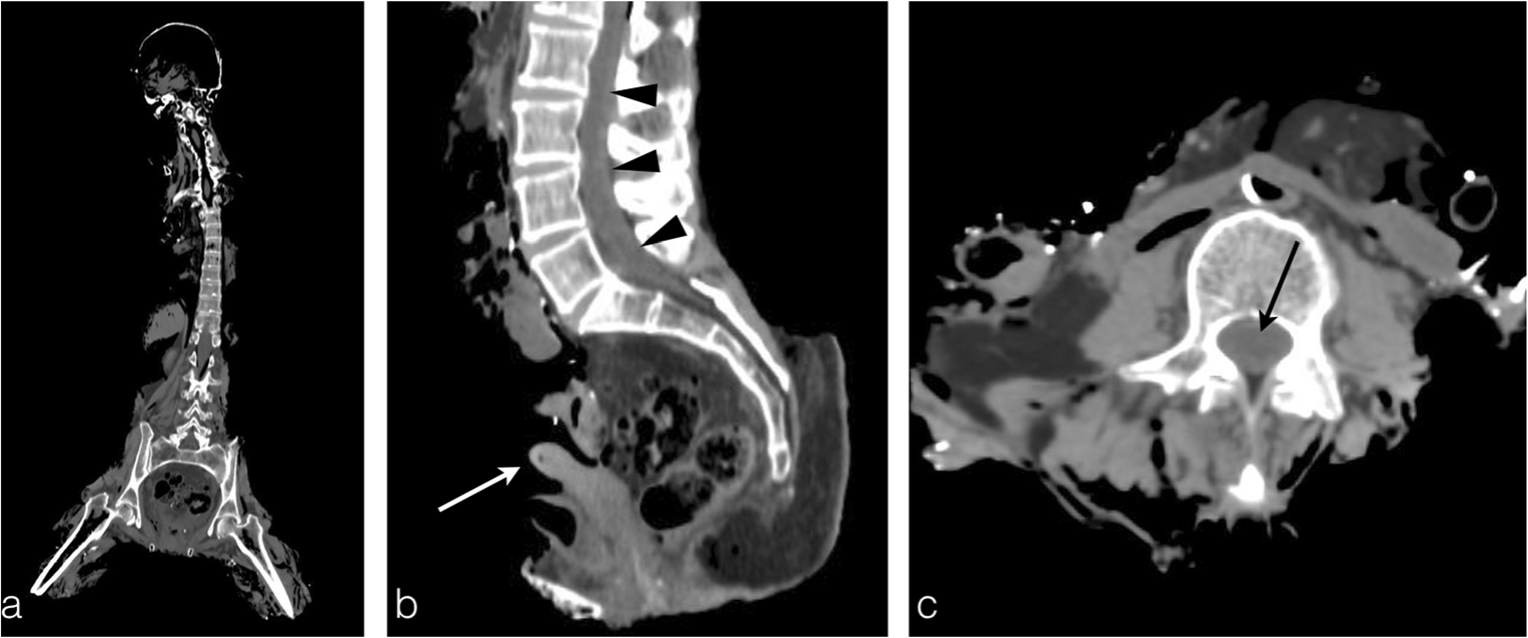
The charred body of a 68-year-old woman, who committed suicide by burning her house. **a** PMCT coronal reconstruction shows an extensively destroyed body, which renders formal identification very challenging. **b** Sagittal reconstruction conveys the presence of a uterus, proving a female victim (*white arrow*). The spinal cord is preserved despite the advanced state of tissue destruction (*arrowheads*). **c** Axial plane image showing that the spinal canal presents no heat disruption (*black arrow*), preserving the spinal cord and meninges for DNA sampling

In more extreme cases, corpses are in such a damaged state that the victim’s secondary sexual characteristics are no more distinguishable and the gender can no longer be defined. Nevertheless, the deep organs are relatively preserved from the heat by the abdominal wall or the peritoneum. Thus, the uterus or prostate are most of the time present on PMCT, allowing the radiologist to determine the subject’s gender (Fig. 4a).

In these particular extreme cases, the forensic team may encounter difficulties in collecting bone samples. They usually collect the femoral bone to proceed to the DNA analyses [5]. Yet, when the corpse displays extensive thermal destruction, samples can be collected from the spinal cord or, even better, from the dura mater, which is well known to better resist thermal injuries because of underlying anatomical attributes [6, 7].

It is interesting then for the radiologist to point out the preserved structures that remain available for DNA sampling in order to assist the identification process (Fig. 4b-c).

## Fluid sampling

One of the crucial tasks during an autopsy is the collection of fluid samples for toxicological screening. This step is particularly important in the case of a charred body; in addition to the usual toxicological screening, the forensic pathologist would seek to ascertain the percentage of serum carboxyhaemoglobin (COHb) in order to determine whether death occurred before or during the fire [8].

The success of this procedure depends on the availability of biological fluids directly related to the burning level of the body. A detailed imaging analysis of the PMCT provided by an expert radiologist can be extremely useful to point out the possible collecting sites.

**Fig. 5:**
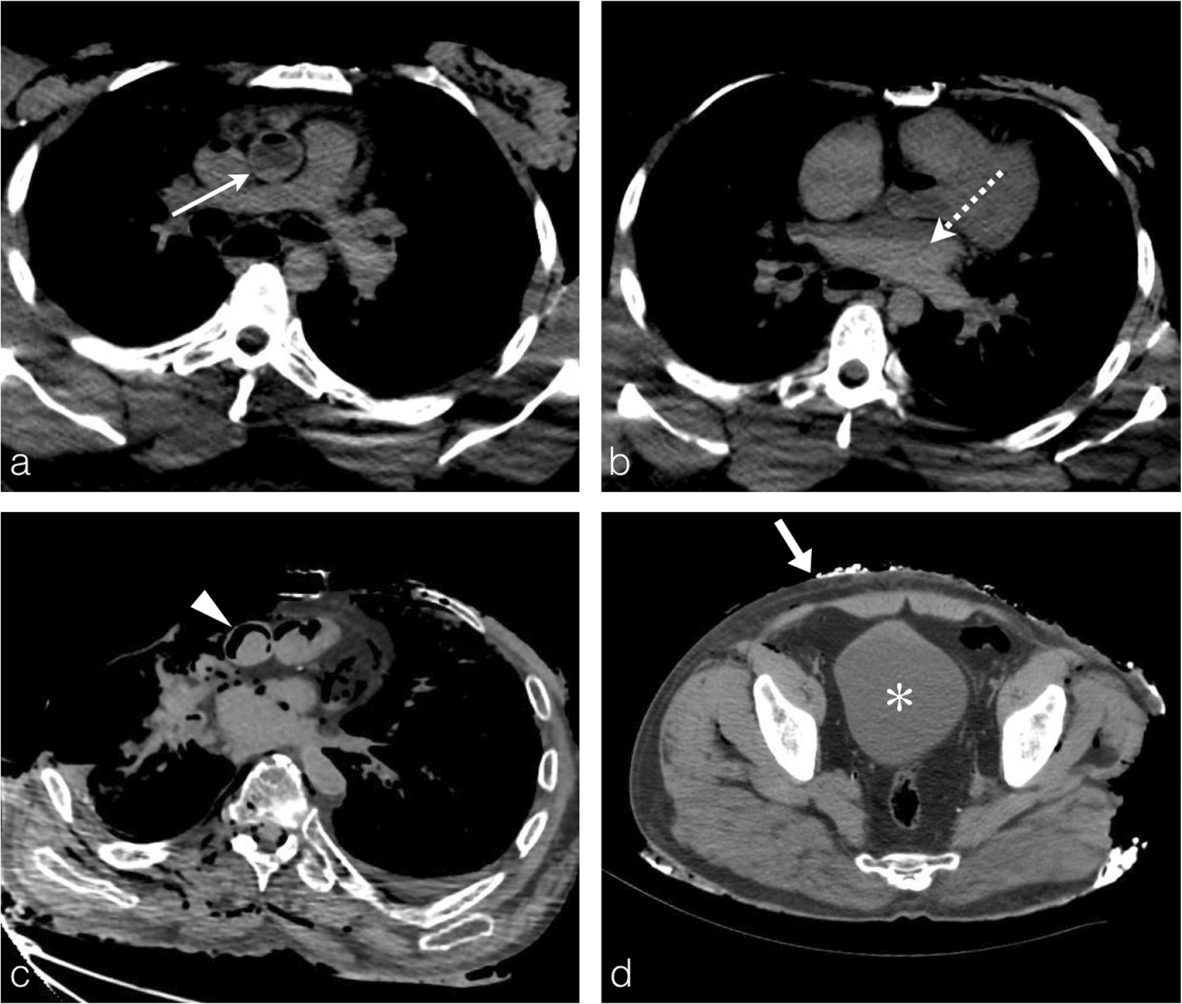
Fluid sampling. **a, b** A 39-year-old man who died in a fire at his home. Axial thoracic CT images viewed in the mediastinal window clearly show a fluid-fluid level in the ascending aorta (*white arrow*) and left atrium (*white dashed arrow*), pointing out non-coagulated blood available for sampling and toxicological analysis. **c** The severely burned body of an 89-year-old man. Axial thoracic CT image showing a thermal pericardial disrupture with exposure of the ascending aorta and cardiac cavities. An hyperdense round image is clearly identified in the lumen of the ascending aorta surrounded by a fine aeric crescent corresponding to a heat-related clot (*arrowhead*). There are also no fluid-fluid levels in the cardiac cavities or mediastinal vessels, suggesting the presence of thermally induced blood coagulation, and hence eliminating the possibility for blood sampling. **d** Same body as **c**: axial plane pelvic CT image showing a full bladder suitable for urine sampling (*asterisk*) through moderate loss of abdominal skin protecting the bladder. Note the asymmetry of fire-related injuries, with the thorax displaying multiple significant chest wall injuries, and the pelvic wall exhibiting thinning and a localised area of hyperattenuation, denoting hyperdensities of burned ashes (*thick white arrow*)

Blood is the preferred medium for this toxicological analysis. The post-mortem circulatory arrest results in an increase in the haematocrit level in the dependent regions of the cadaver [9]. Thus, due to the hypostasis, the PMCT often shows a fluid-fluid level in the great vessels and the cardiac cavities, especially when the mechanism of death is not related to direct blood loss and subsequent hypovolemic shock (Fig. 5a, b). This is a sign of non-coagulated blood, suitable for sampling.

Unfortunately, sometimes the intensity of heat-related injuries can be so substantial to the point of destroying the thoracic wall and pulmonary tissue of the mediastinum. Subsequently, the blood coagulates in the heart and great vessels preventing any blood collection for sampling (Fig. 5c). In this case, other biological fluids may be used, such as urine.

Therefore, the radiological report should always embrace a clear description of the blood status in the heart and great vessels, and precision regarding the presence (or absence) of urine in the bladder (Fig. 5d).

## Cause of death

The main goal of the forensic investigation is to determine the cause of death: is death subsequent to the fire or was the deceased a victim of homicide, and the fire setting being simply an arson to cover it up? The cause of death may also be traumatic and the fire a consequence, which is the case in some car accidents.

Once more, the state of carbonisation complicates the post-mortem autopsy. The charred tissues are difficult to dissect from the bones, and the traumatic fractures could be missed by the macroscopic analysis. In the same way, ballistic foreign bodies as plumb shrapnel or bullets could also be missed during the autopsy.

**Fig. 6:**
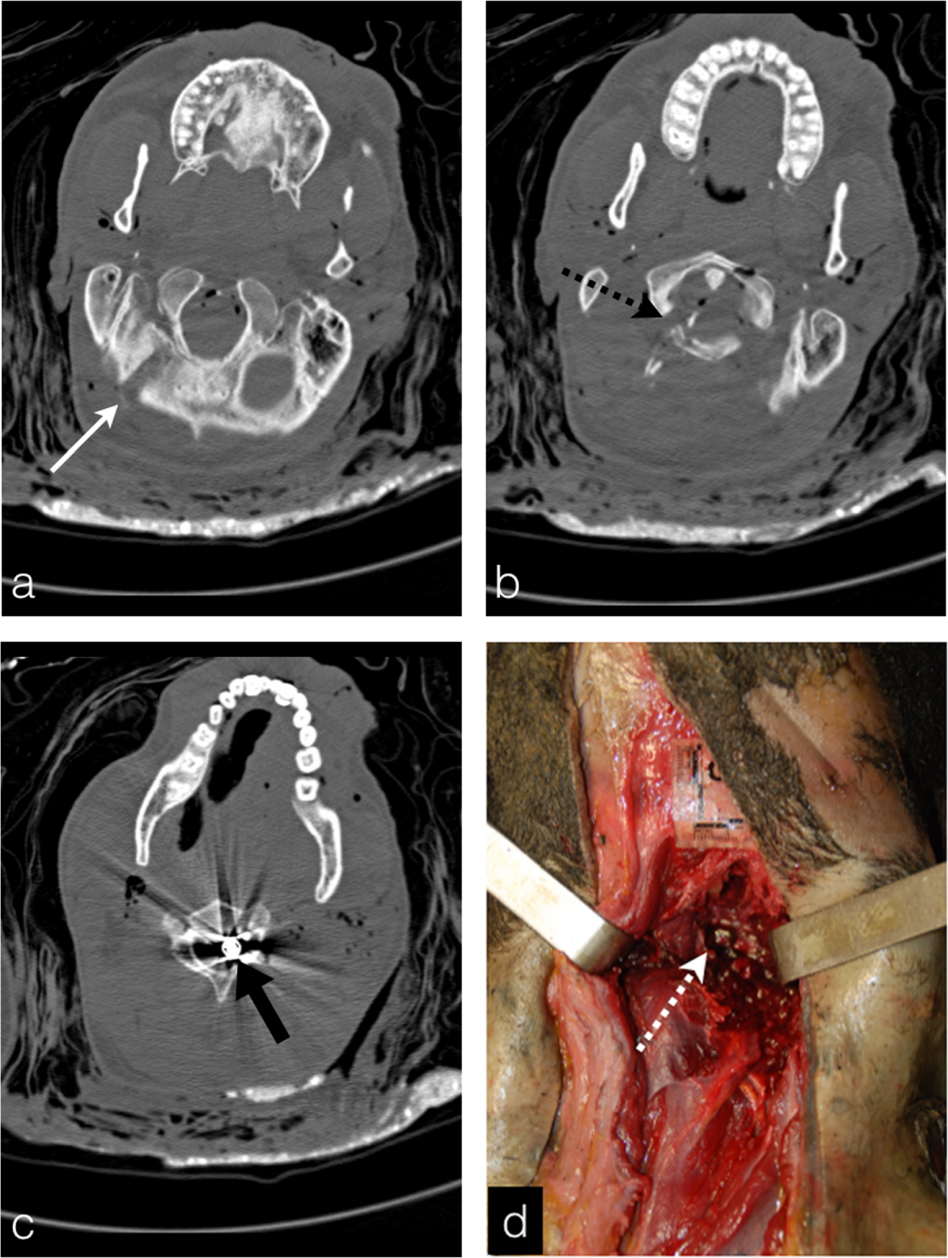
A 32-year-old man who was shot dead, burned and buried in a field. **a-c** Metallic artefact of the bullet within the cervical canal (*thick black arrow*) opposing the second cervical vertebra (C2). View of the gunshot entry wound in the occipital bone (*white arrow*) with a downward trajectory through the first cervical vertebra, which is fractured (*black dashed arrow*). The bone fractures allow for a reliable bullet trajectory reconstruction. **d** Autopsy photograph of the same body guided by the PMCT with the bullet in the cervical canal (*white dashed arrow*)

**Fig. 7:**
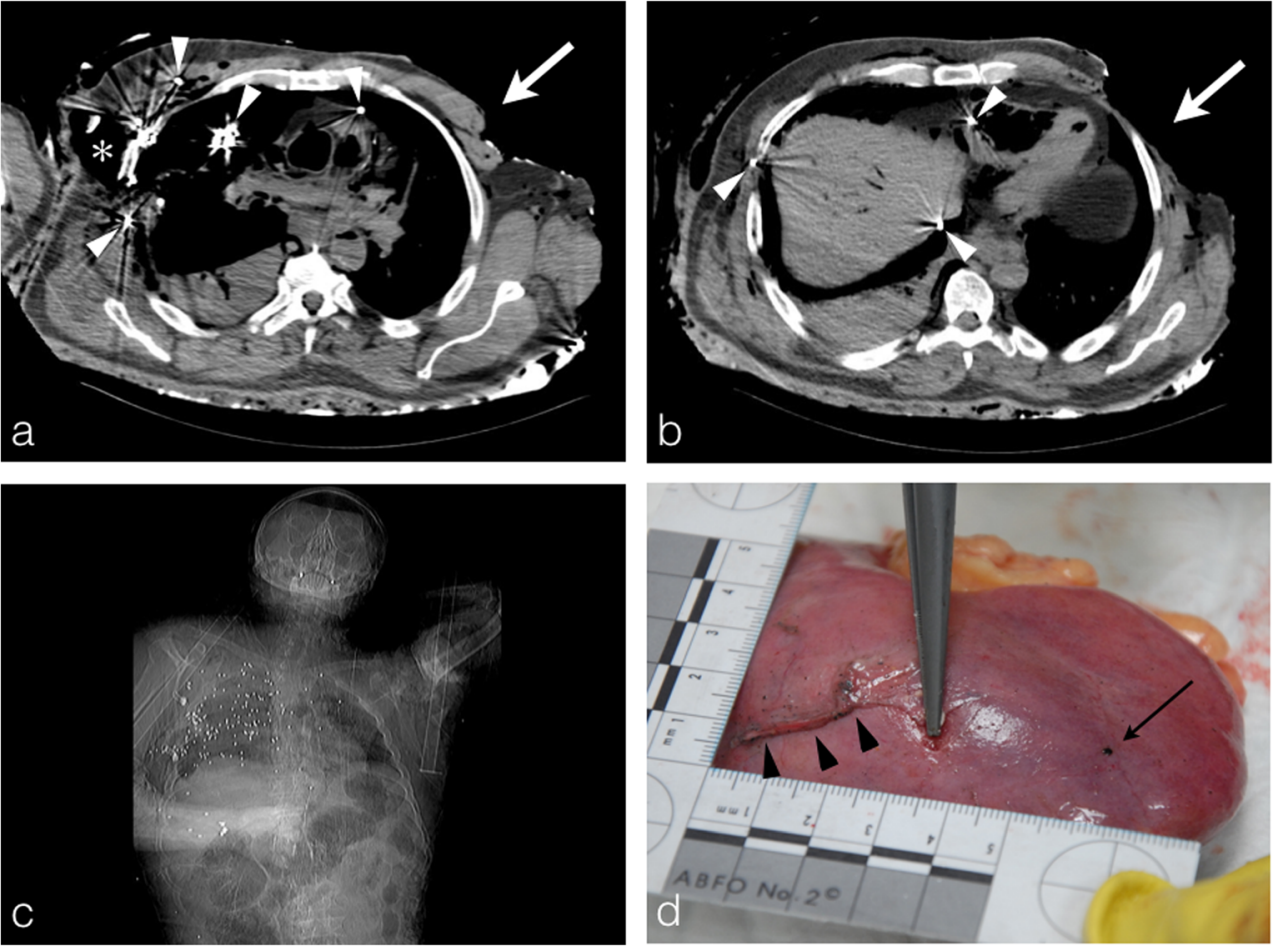
A 69-year-old man, shot and burned in his house. **a, b** Axial PMCT planes show several shrapnel fragments in the right thoracic, the right lung, the mediastinum and the abdomen specifically around the liver (*white arrowheads*). Note the important soft-tissue loss of the right laterothoracic wall (*asterisk*) corresponding to the entrance wound. The body was more exposed to the fire on its left side with heat-related skin loss (*thick white arrows*), preserving the entrance wound (important issue for forensic evaluation). **c** PMCT scout image showing the multiple shrapnel fragments as punctiform hyperdensities (about 200). **d** Liver autopsy photograph of the same body showing a linear cut due to the shrapnel fragment course through the right liver (*black arrowheads*) with shrapnel fragment lying on the right liver surface (*black arrow*). The shrapnel localisation by PMCT guided the autopsy analysis

Due to its sensibility to metallic artefacts, PMCT is a major asset in gunshot injuries to depict the ballistic foreign bodies and their locations in the burned cadavers [10]. PMCT is discerning between entrance and exit wounds, identifying the projectile trajectory and helping to specify the death mechanism [11] (Fig. 6). There may be plenty of bullet fragments inside the body, especially in cases of hunting rifles and lead shots, and they must be counted and mentioned in the final radiological report. In this case, PMCT shows several millimetric hyperdensities surrounded by metallic artefact (Fig. 7).

**Fig. 8:**
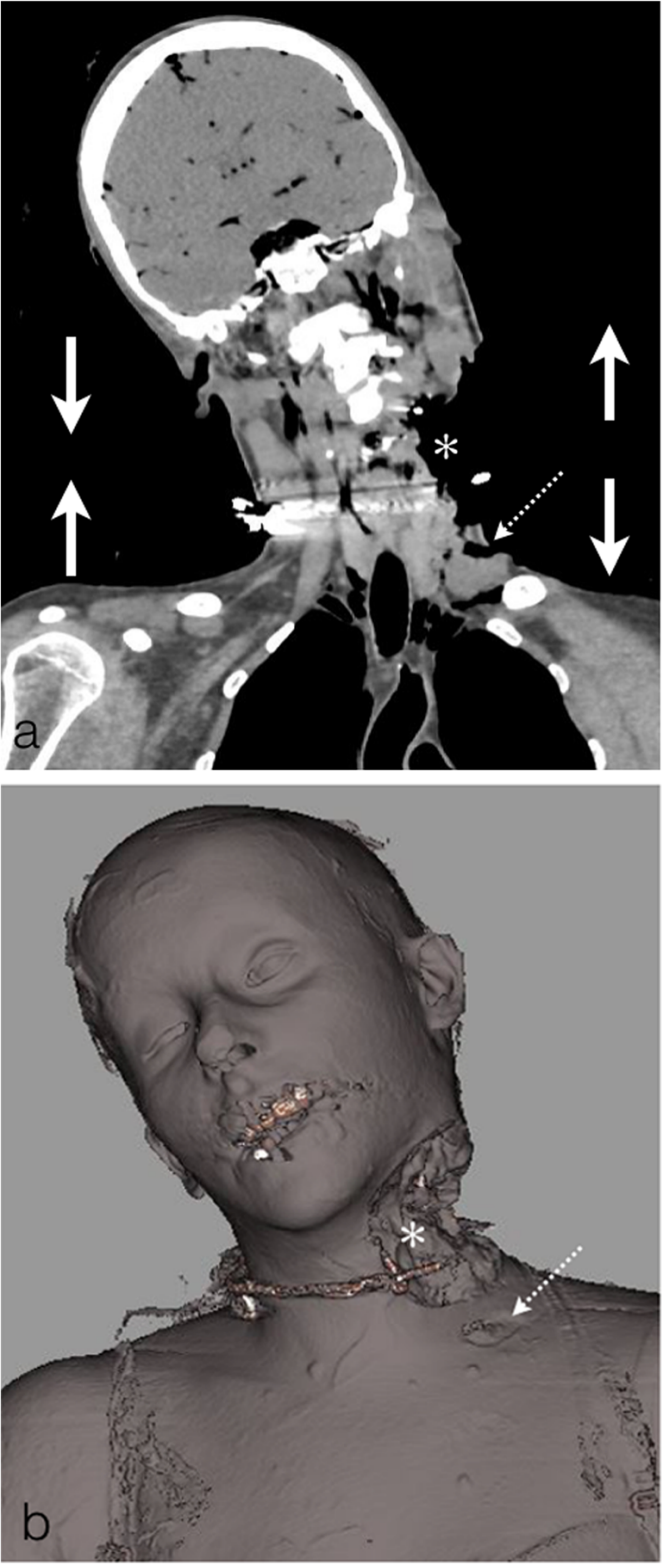
A 46-year-old woman who was stabbed in the neck, died of carotid haemorrhage and then was partially burned in a fire initiated with accelerant substances (criminal intent). **a** PMCT coronal reconstruction shows a deep laterocervical wound with clean edges (*asterisk*). The head position amplifies the distance between the wound edges (*thick arrows*). Note the second stab wound next to the left subclavicular fossa (*dashed arrow*). **b** Volume rendering reconstruction shows better the clean edges of the wound

Suspicious skin lesions that could evoke a bladed weapon homicide must also be examined. In such cases, contrary to thermal skin injuries, stab wounds exhibit clean edges with evidence of subcutaneous emphysema surrounding the linear cut lines on PMCT (Fig. 8).

**Fig. 9:**
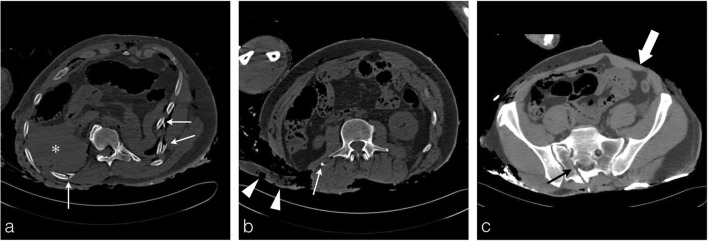
A 58-year-old man who died in an ultralight aircraft crash with a secondary fire setting: the PMCT shows a mixture of traumatic and thermal lesions. The traumatic lesions are severe (death seems related to trauma). **a** PMCT shows rib fractures on unexposed bones classified as traumatic fractures (*white arrows*). Skin burn lesions and skeletal muscles exposition are seen on the right postero-lateral chest wall. Note the post-traumatic right haemothorax (*asterisk*) consequent to the rib fracture (*white arrow*). **b** Major soft-tissue burn lesions with secondary detachment of the wall (*white arrowheads*). Note also a traumatic right transverse process fracture of L2 vertebrae (*thin white arrow*) at the abdominal level. **c** Right sacral traumatic fracture (*black arrow*). Please note the left anterior abdominal wall thermal lesion with intestinal exposure and doubt concerning peritoneal continuity (*thick white arrow*)

In other circumstances such as car accidents, the victim could first decease from severe traumatic injuries and the fire breaks out thereafter. The PMCT then displays post-traumatic lesions similar to a living polytraumatic patient in addition to heat-related lesions (Fig. 9). Traumatic fractures are unlike thermal fractures (*see* the section “Bones”).

## Characteristic burn-related lesions

### Overall state of the body

**Fig. 10:**
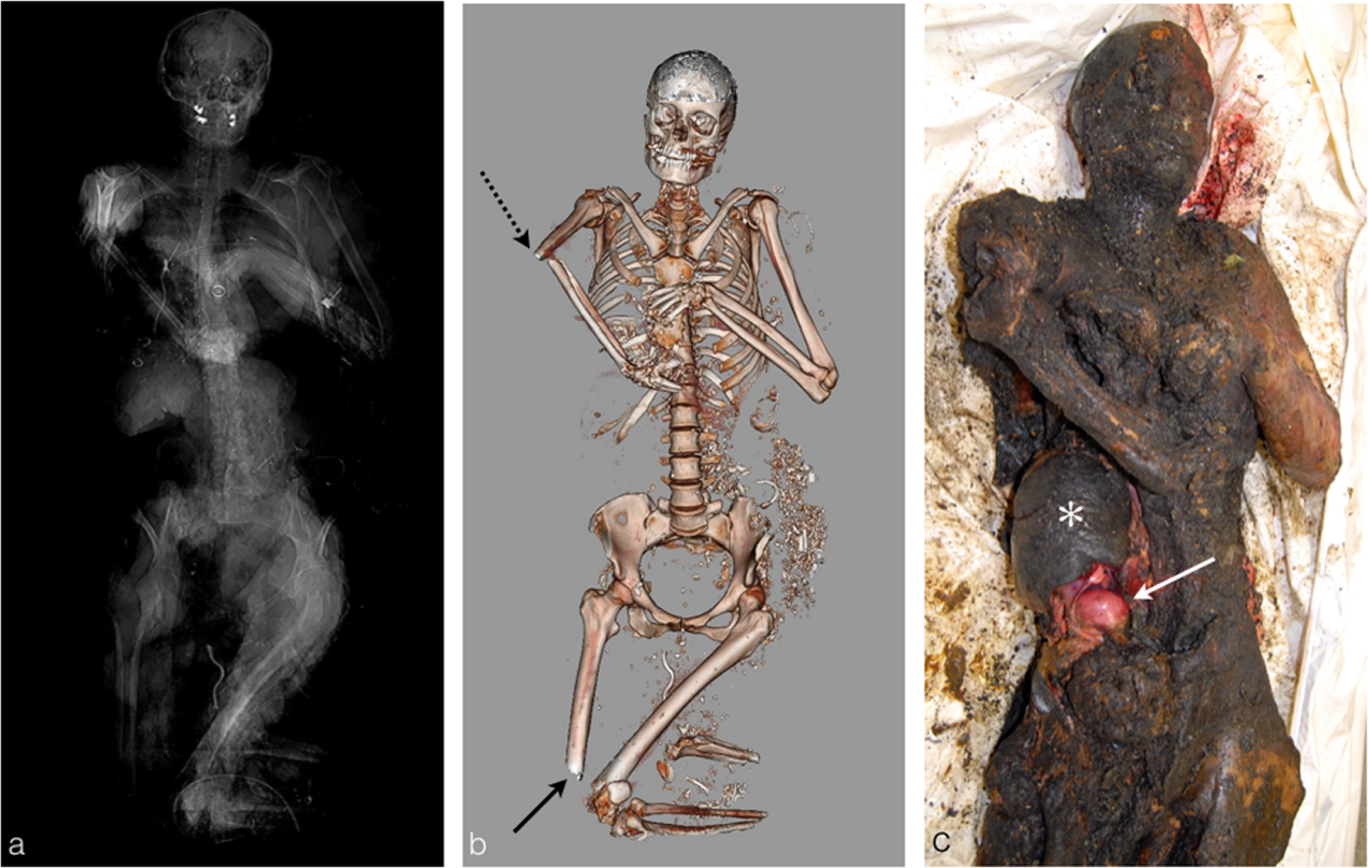
The burned body of a 35-year-old woman who was killed in a public-road car crash. **a** PMCT scout view and **b** volume rendering reconstruction showing the characteristic triple flexion of the left upper limb due to muscle thermal retraction: pugilistic attitude consisting of an anteromedial flexion of the humerus on the body, flexion of the forearm on the arm and flexion of the wrist on the forearm. Additionally, notice the typical thermal fracture of the right humerus (*black dashed arrow*) and the right femur of the lower extremity (*black arrow*). **c** Autopsy photograph of the same body showing a perfect concordance with volume rendering reconstruction of the triple flexion of the upper left limb. The image also shows the extreme loss of abdominal wall soft tissues with exposure of the burned liver (*white asterisk*) and preserved right kidney (*white arrow*)

The fire starts by burning off the skin and the soft tissues resulting in skeletal muscle exposure. Exposed muscles then contract and shrink because of the heat, leading to a flexion deformation of the limbs. The upper limbs present a characteristic triple flexion: the body takes the position of a boxer holding his hands in front of him called the pugilistic attitude [12]. Such thermal consequences on the burned body’s overall state appear quickly after exposure to the fire, in about 10 min [3], and are easily shown on the PMCT, especially on the scout view and volume rendering reconstructions (Fig. 10).

### Head

Because of the trivial thickness of the head’s soft tissues and the weak protection on it (absence of clothes), the face and the skull are the predominant areas of heat-related lesions in a burned victim. These injuries are typical, resulting from the effect of prolonged exposure to the heat. Depending on the duration of fire exposure, different patterns are described in forensic medicine [3].

The radiologist must recognise them and distinguish them from the other common post-mortem changes and, more importantly, from other features that could suggest ante-mortem physical trauma or disease.

The first injuries occur on the superficial tissues and are macroscopically seen as skin lacerations. They are seen quickly after the beginning of fire exposure and are laboriously detected by PMCT.

Then, loss of soft tissues is observed, which is often responsible for large-bone exposure.

**Fig. 11:**
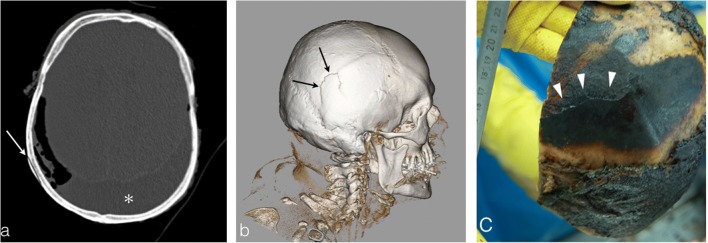
Skull images of a 53-year-old man who died by asphyxia and was found in the procubitus position in his burned house. **a** Linear, fine, superficial fracture of the outer table (*white arrow*) in an area of a thermal soft tissue loss. Notice the associated thermal epidural haematoma (*asterisk*). **b** Volume rendering reconstruction depicts more easily this fine and superficial fracture (*black arrows*). **c** The autopsy picture shows perfect concordance with the PMCT images with a linear outer table fracture of the right temporal bone (*white arrowheads*). Note the soft tissue damage with bone exposure

**Fig. 12:**
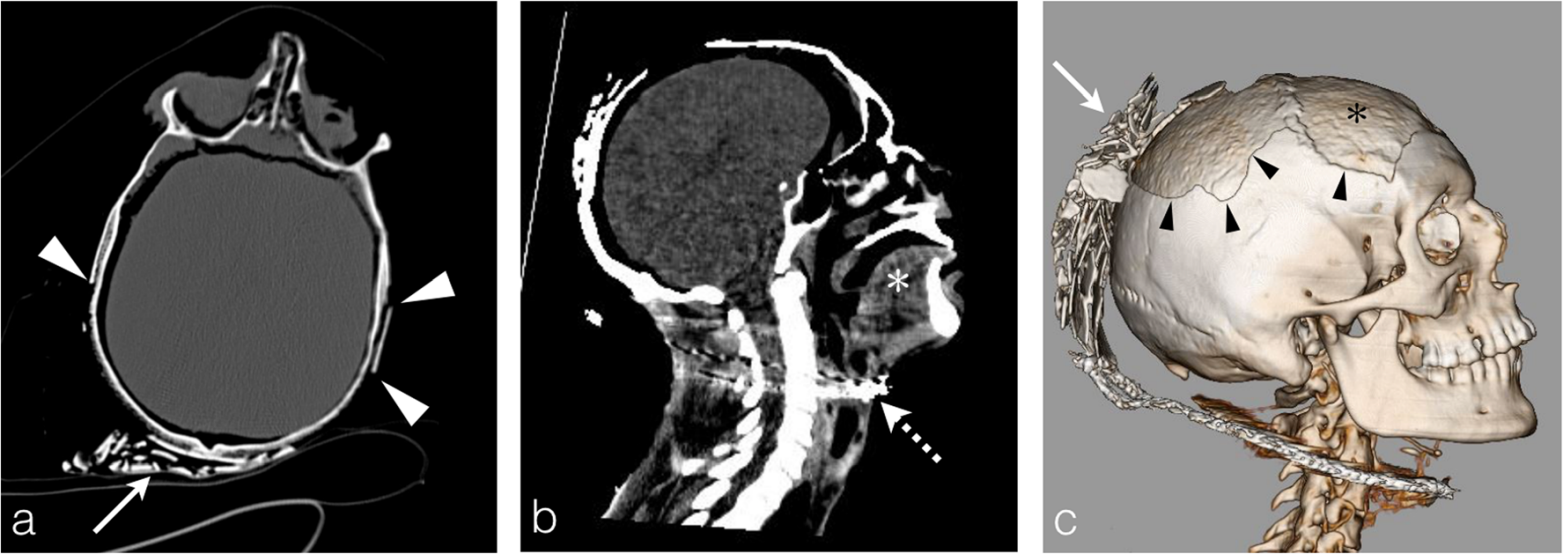
The burned skull of a 32-year-old man who died in the context of carbon monoxide poisoning in a fire at his home. **a** Please note the outer table heat fractures by delamination (*white arrowheads*) with multiple bone fragments in the surrounding scalp (*white arrow*). **b** Sagittal reconstruction shows the characteristic tongue protrusion related to death by asphyxia (*white asterisk*). Note the metallic artefact of a necklace which can permit body identification (*white dashed arrow*). **c** On volume rendering reconstruction, the irregular edges of outer table heat fracture (*black arrowheads*) with inner table integrity (*black asterisk*) are easily seen

Under the heat effect, bone lesions begin to appear, visible on PMCT as fine, linear, outer table fracture lines (Fig. 11). The next step is the complete delamination of the outer table in multiple fragments, while the inner table remains intact (Fig. 12).

In parallel to these bone lesions, the heat is also responsible for changes inside the cranial cavity.

**Fig. 13:**
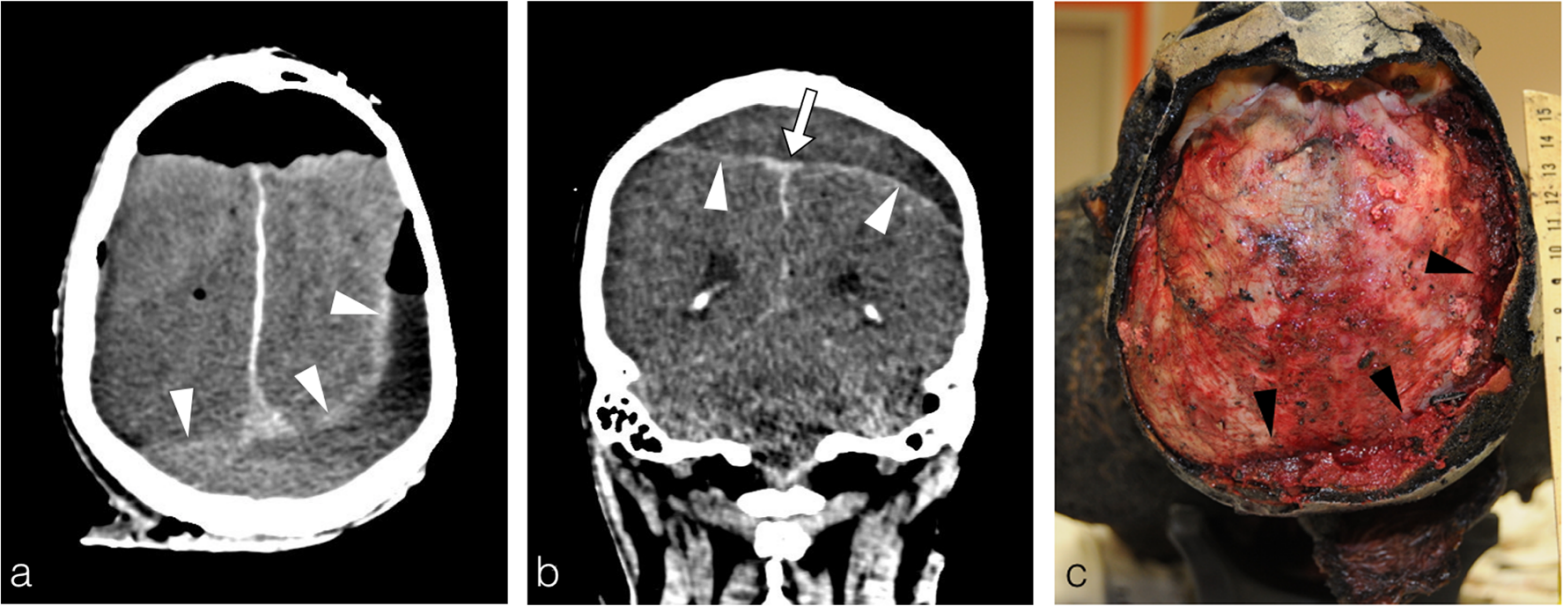
The charred body of a 71-year-old man found in a burning house following a homicide with gunshots, where approximately 200 shrapnel fragments were identified in the chest region (not shown here). **a, b** Typical thermal epidural haematoma: crossing the midline, crescent shaped, with a subdural appearance (*arrowheads*). The coronal reconstruction shows detachment of the sagittal superior sinus (*thick white arrow*). There were no shrapnel fragments in the skull. **c** Autopsy confirmed a thermal epidural haematoma with perfect agreement with the PMCT images (*black arrowheads* dura mater)

The increasing temperature inside the skull causes dura mater retraction, which exfoliate from the bone and leads to blood exudation from the venous sinuses within the epidural space. Thereafter, under the continuing heat, this blood collection coagulates and forms the characteristic heat epidural haematoma, which is one of the most frequent heat-induced lesions found in burned bodies. The radiologist must distinguish this heat-haematoma from an epidural haematoma caused by blunt trauma. The heat-haematoma is low density and crescent shaped, similar to a subdural haematoma but often crossing the midline and detaching the venous sinus (Fig. 13), whereas an epidural haematoma caused by trauma is highly dense, convex and lens shaped [2–4]. The pathogenesis easily explains these differences: the heat-haematoma results from the exudation of blood from the venous sinuses, while a post-traumatic epidural haematoma is caused by arteriovenous injuries due to a skull fracture secondary to an external blow.

**Fig. 14:**
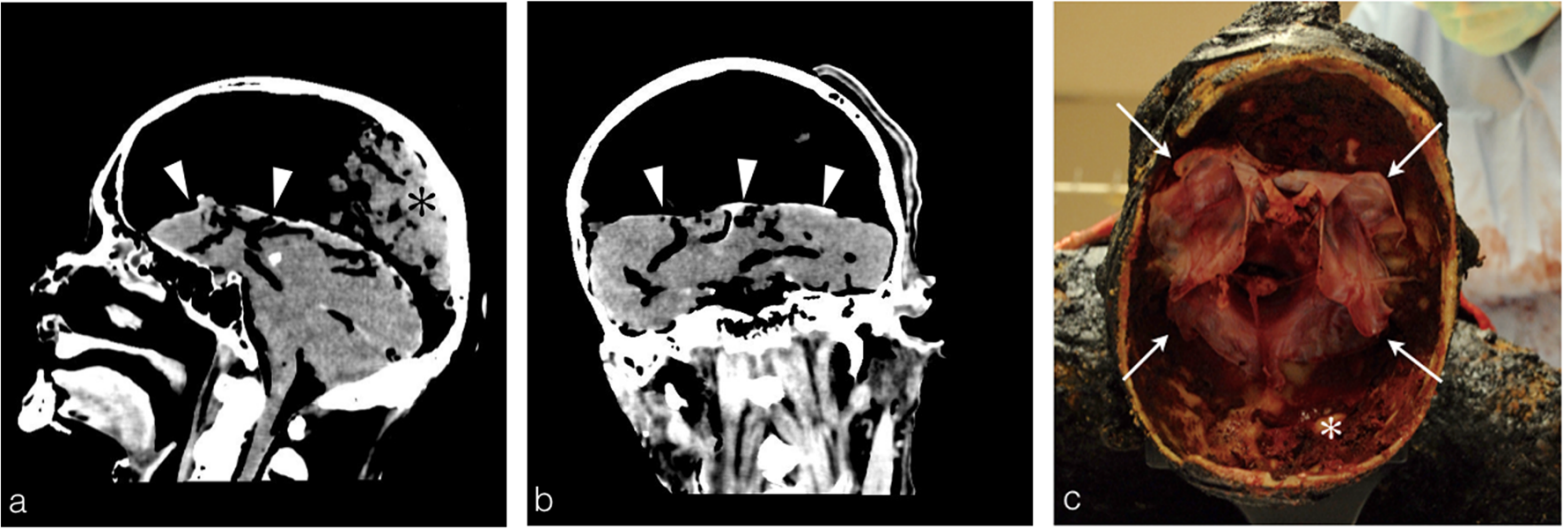
Head PMCT of a 73-year-old man found burned in a home fire. **a** The sagittal reconstruction shows the retraction of the cerebral hemispheres towards the base of the skull (*white arrowheads*) with heterogenous posterior heat epidural haematoma in declivitous position (*black asterisk*). Note the integrity of the skull (no fracture, no cerebral matter loss). **a** The retraction of the dura mater is more evident on the coronal reconstruction with a complete detachment from the skull arch (*white arrowheads*). **c** Autopsy photograph: superior view after skull opening showing retracted cerebral matter towards the skull base (*white arrow*) and posterior heat epidural haematoma (*white asterisk*)

In extreme cases, a complete detachment of the dura mater of the skull arch with retraction of the cerebral hemispheres towards the base of the skull can be observed (Fig. 14).

**Fig. 15:**
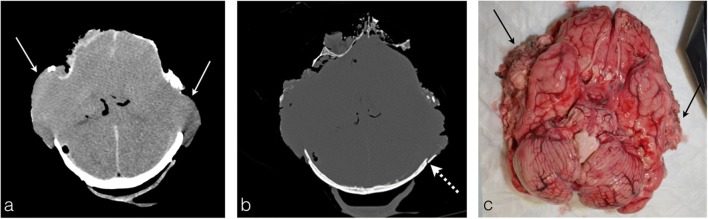
The skull of a 39-year-old man who died by asphyxia in an accidental fire at his home. **a** PMCT shows bilateral meningeal disruption with brain herniation (*white arrows*). **b** In the bone window, notice the important skull destruction with heat fracture and loss of temporal bones (*dashed white arrow*). **c** The autopsy photograph shows the burned herniated brain, which assumes a “cauliflower” or “mushroom” appearance (*black arrows*)

In areas where heat acted locally and severely on the skull leading to bone disrupture, the dura mater can still remain intact. On the contrary, sometimes the dura mater is disrupted without being directly burned and heat-induced brain herniation in the epidural spaces develops (Fig. 15a, b). The pathogenesis is still unknown, but a boiling mechanism due to the local thermal effect is postulated for an increase in pressure causing a split of the dura mater. The cerebral tissue is then allowed to exit the skull and to burn under the heat effect [13]. The forensic pathologist can observe the macroscopic appearance of the herniated brain, which assumes a “cauliflower” or “mushroom” appearance (Fig. 15c).

**Fig. 16:**
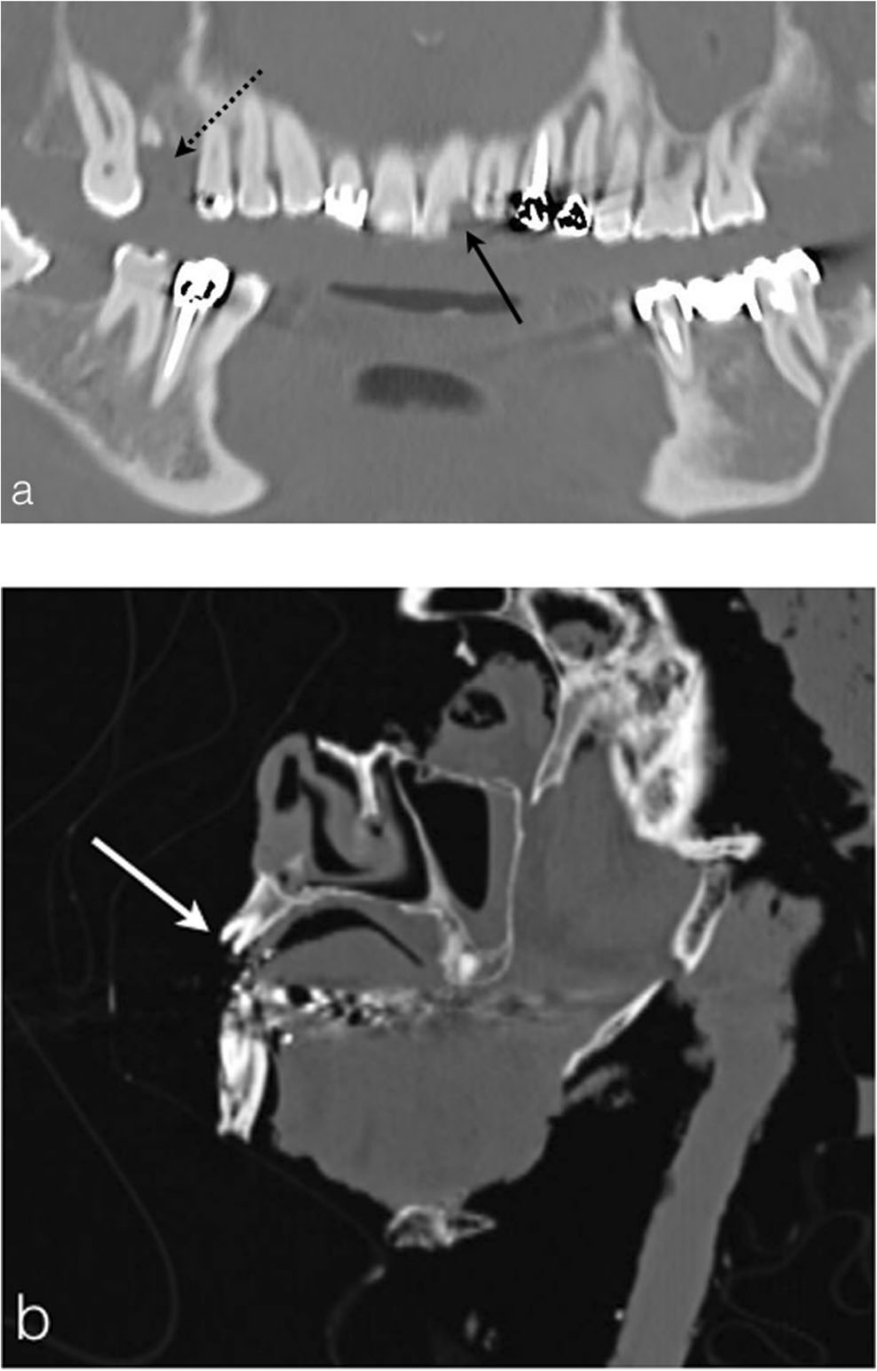
**a** Dentascan reconstruction of a 70-year-old woman who committed suicide by immolating herself, showing fracture with detachment of the no. 21 incisor (*black arrow*) and the fracture with tooth avulsion of the no. 16 molar (*black dashed arrow*). **b** Head PMCT sagittal reconstruction of a 32-year-old man found burned on his sofa, showing the crown detachment of the no. 21 incisor (*white arrow*)

The fire may also damage the teeth, complicating the identification process of the victim. PMCT may show fine tooth fissure (no displacement), tooth fracture (with displacement) (Fig. 16 b) and, for the most severe tooth injury, a crown detachment (Fig. 16a). The heat also may have an impact on the alveolodental bone, which can present thermal fractures, leading to an alveolodental space enlargement with possible partial or complete tooth avulsion [14]. As all medical implants, dental filling material should be mentioned in the PMCT final report for victim identification purposes.

### Airways and chest

When the fire starts, the victim—whether conscious or not—will quickly inhale smoke and soot from the fire that will deposit in the deep respiratory tract and the oesophagus. The soot is unfortunately not detected on the PMCT but is visible at the autopsy as black deposits on the mucous membranes.

However, other essential signs are viewable on the PMCT and may allow for the deduction that the deceased was breathing, and hence alive, at the beginning of the fire.

The first sign of asphyxia is the tongue protrusion through the dental arches and the lips—more easily identified on the sagittal reconstructions (Fig. 12) [15].

**Fig. 17:**
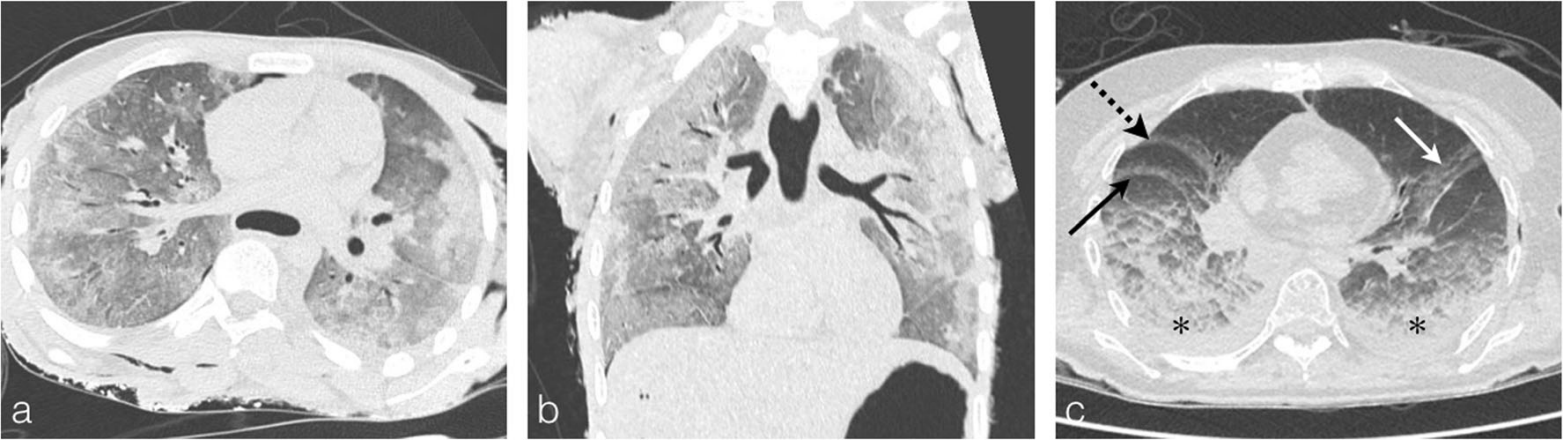
**a, b** A 40-year-old man who died by carbon monoxide poisoning in a home fire. Note the ground-glass opacities of pulmonary oedema, better visualised on the coronal reconstructions. These lesions are diffuse, reaching the whole pulmonary lobes, and different from livor mortis pulmonary lesions due to hypostasis. **c** A 32-year-old man who was shot dead in the head and burned afterwards. Ground-glasses opacities in the posterior and dependent portions of lungs (*asterisk*), against the left oblique fissure (*white arrow*), the right oblique fissure (*black arrow*) and the horizontal fissure (*black dashed arrow*) corresponding to characteristic livor mortis lesions not to be confused with heat or asphyxia lesions

Thereafter, the inhalation of hot gases causes tract mucosal damage inducing pulmonary oedema. This oedema assumes the appearance of a classical pulmonary oedema with diffuse ground glasses opacities reaching the whole pulmonary lobes (Fig.17). Such lesions need to be differentiated from the lung lividities due to hypostasis where the ground glass opacities are declivitous and posterior in location, against the pulmonary fissures and sparing the upper zones with a density gradient [16].

### Abdomen

The intra-abdominal organs are long-time preserved from the fire via the multiple soft-tissue layers protecting them: first the skin and subcutaneous fat, then all the abdominal wall muscles, and finally the peritoneal fascia.

**Fig. 18:**
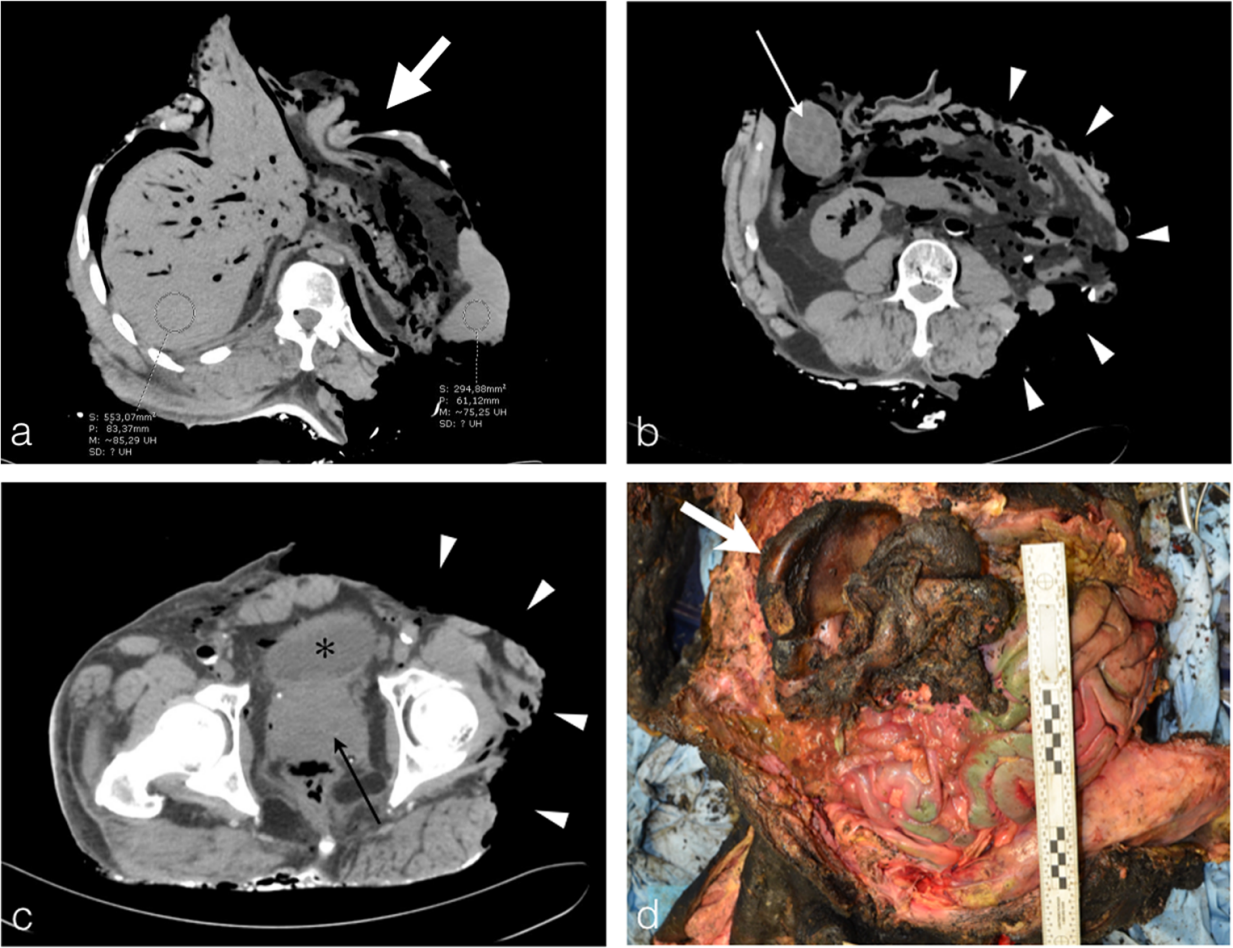
The unidentified charred body of a 70-year-old man found in a burning house. **a, b** Axial plane PMCT images show large soft-tissue loss on the body’s left side with peritoneal interruption and evisceration (*thick white arrows*). Note the multiple, rounded, low-density images in the gall bladder (*thin white arrow*) corresponding to cholesterol lithiasis in autopsy. **c** The visualisation of the prostate (*black arrow*) confirms a male victim. Notice that despite the left abdominal wall and soft tissue burning, it is difficult to confirm the peritoneum’s integrity on the lower abdominal part (*arrowheads*). It is also important to report the availability of urine in the bladder for fluid sampling and toxicological analysis (*asterisk*). **d** Autopsy picture of the same body. Despite its burned macroscopic aspect (*white dashed arrow*), the liver density remains unchanged on the PMCT axial plane (85 HU) (**a**)

The peritoneum’s integrity could be difficult to clarify on the PMCT because of its thinness. However, the visualisation of evisceration of intestinal or other intra-abdominal organs definitely proves its interruption (Fig.18). This intestinal evisceration is a delayed heat-induced consequence occurring after approximately 30 min of fire exposure [3]. The intra-abdominal organs, especially the liver, do not show spontaneous density modifications, regardless of their burning macroscopic state (Fig. 18a-c).

### Bones

As we see, the heat is quickly responsible for skin and soft-tissue thermal lesions. Subsequently, the heat leads to muscle retraction and bone exposure.

As for the skull, the first bone heat-related lesions are linear, fine fractures on the exposed bone called thermal cortical fractures.

**Fig. 19:**
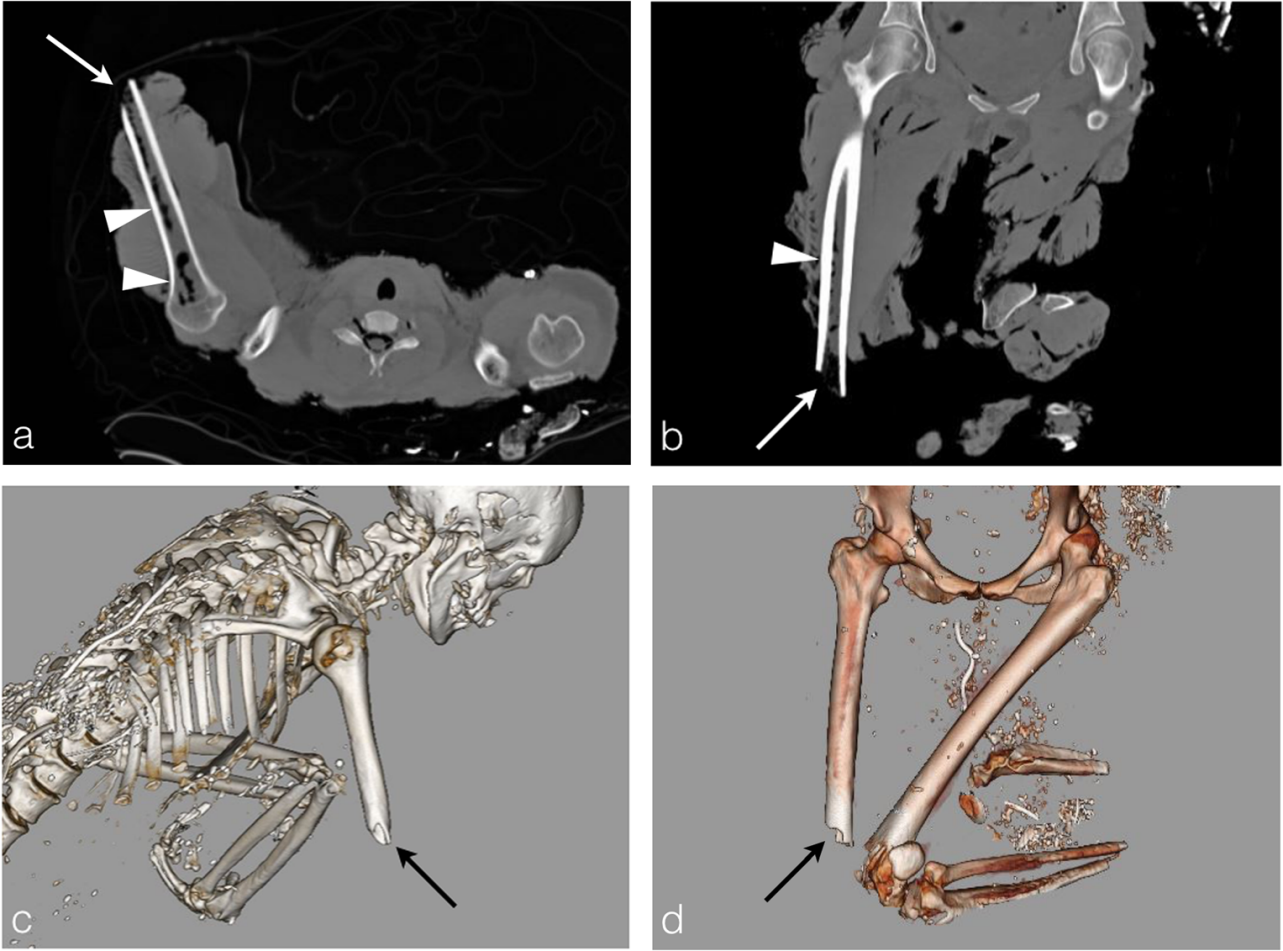
A public-road car-crash victim: a 35-year-old woman whose body was burned. **a, b** Smoothly marginated fractures uncovered by skeletal muscle: thermal amputations of the extremities (*white arrows*). Please note the mottled lucencies of marrow spaces upon the heat exposed side with accompanying soft-tissue retraction (*arrowheads*). **c, d** Typical appearance of “flute mouthpiece” on volume rendering reconstructions (*black arrows*) without any argument for traumatic fracture (no sharply angulated margins). Note equally the pugilistic attitude of the left arm (triple flexion) on volume rendering reconstruction (**c**). Typical overall state of the body in a fire-related death

When the limb extremities are completely burned, PMCT shows thermal amputation with transverse, smoothly marginated fractures that are uncovered by soft tissue (Fig. 19a-c). This bone amputation takes the characteristic appearance of a “flute mouthpiece”, more easily viewable on volume rendering reconstructions (Fig. 19b-d).

These thermal fractures and amputations are different from the traumatic ones, which are most of the time covered by soft tissue and presenting clean, angulated margins, sometimes with evident comminution [12].

The heat is also responsible of internal bone structure modifications, especially of the bone marrow. PMCT may show a pattern of mottled lucencies in bone marrow spaces, only where the bone has been directly exposed to the fire (Figs. 2b-19). In parallel with the forensic investigation, these bone marrow lesions could help to clarify the heat source and fire outbreak localisation.

## Conclusions

PMCT is currently an accessible and contemporary tool for forensic investigations. This pictorial essay highlights the key themes for a practical approach in the scenario of PMCT investigating burned victims and the particularities of PMCT reports, which should contain the available identification elements and technical clues for the forensic team. The semiological analysis must be made according to the fire context and any discrepancies must be brought to the pathologist’s attention in order to guide the autopsy procedure.

## References

[1] Thali MJ, Yen K, Schweitzer W (2003). Virtopsy, a new imaging horizon in forensic pathology: virtual autopsy by postmortem multislice computed tomography (MSCT) and Magnetic resonance imaging (MRI)—a feasibility study. J Forensic Sci.

[2] Dirnhofer R, Jackowski C, Vock P, Potter K, Thali MJ (2006). VIRTOPSY: minimally invasive, imaging-guided virtual autopsy. Radiographics.

[3] Bohnert M, Rost T, Pollak S (1998). The degree of destruction of human bodies in relation to the duration of the fire. Forensic Sci Int.

[4] Thali MJ, Yen K, Plattner T (2002). Charred body: virtual autopsy with multi-slice computed tomography and magnetic resonance imaging. J Forensic Sci.

[5] Butler J (2005) Forensic DNA typing biology, technology, and genetics of STR markers, 2nd edn. ACADEMIC Press INC, England

[6] Motani H, Sakurada K, Akutsu T (2006). Usefulness of dura mater in providing DNA samples for identifying cadavers. J Forensic Sci.

[7] Harcke HT, Monaghan T, Yee N, Finelli L (2009). Forensic imaging-guided recovery of nuclear DNA from the spinal cord. J Forensic Sci.

[8] Schwerd W, Schulz E (1978). Carboxyhaemoglobin and metheamoglobin findings in burnt bodies. Forensic Sci Int.

[9] Jackowski C, Thali MJ, Aghayev E (2006). Postmortem imaging of blood and its characteristics using MSCT and MRI. Int J Legal Med.

[10] Flach PM, Ampanozi G, Germerott T (2013). Shot sequence detection aided by postmortem computed tomography in a case of homicide. J Forensic Radiol Imaging.

[11] Harcke HT, Levy AD, Abbott RM (2007). Digital radiographs (DR) vs multidetector computed tomography (MDCT) in high-velocity gunshot-wound victims. Am J Forensic Med Pathol.

[12] Levy AD, Harcke HT, Getz JM, Mallak CT (2009). Multidetector computed tomography findings in deaths with severe burns. Am J Forensic Med Pathol.

[13] Kondo T, Ohshima T (1994). Epidural herniation of the cerebral tissue in a burned body: a case report. Forensic Sci Int.

[14] Auffret MD, Laurent PE, Saccardy C (2014). Contribution of post-mortem multidetector computed tomography of the head and neck to the forensic evaluation of severely burned bodies. J Neuroradiol.

[15] Bernitz H, Van Staden PJ, Cronjé CM, Sutherland R (2014). Tongue protrusion as an indicator of vital burning. Int J Legal Med.

[16] Hishida M, Gonoi W, Okuma H (2015). Common postmortem computed tomography findings following traumatic death: differentiation between normal postmortem changes and pathologic lesions. Korean J Radiol.

